# Ultrathin Small Outline Package Key Techniques for High-Speed Chips with Multi-Leads

**DOI:** 10.3390/mi15081029

**Published:** 2024-08-13

**Authors:** Lijun Zhang, Wenqiang Dang, Yongshun Wang, Jinbing Zhang

**Affiliations:** 1 School of Electronic and Information Engineering, Lanzhou Jiaotong University, Lanzhou 730070, China; wangysh@mail.lzjtu.cn; 2 School of Electronic Information and Electrical Engineering, Tianshui Normal University, Tianshui 741001, China; dangwenqiang@tsnu.edu.cn; 3 Engineering Research Center, Ministry of Education on Integrated Circuit Packaging and Testing, Tianshui 741001, China; jinbing.zhang@ht-tech.com; 4 Tianshui Huatian Science and Technology Co., Ltd., Tianshui 741000, China

**Keywords:** package, high-speed chip, wire bonding, base island, plastic seal

## Abstract

The key technologies for the ultrathin small outline package (TSOP) of large-sized high-speed chips have been designed and developed in this paper. The designing techniques, such as a 25 µm precise positioning dice attaching technique, a lead frame unit structure without a base island, and a lead co-plane layout inside the frame, were developed. The TSO package outline with a large number of leads, a frame unit arrangement, and a frame distribution with a base island and without one were improved. The technological problems, including the reduction in thickness, wafer cutting, chip sticking bonding, and plastic sealing, were successfully solved. The designed large-sized package products have many advantages, such as high availability, low cost, high reliability, and a short production cycle. This package technique can be widely used in various intellectual application regions.

## 1. Introduction

With the development of integrated circuits focused on miniaturization, high reliability, low cost, high velocity, and multi-chip combinations, it is necessary to realize thinness, short delay times, and the high availability of the IC lead frame materials to satisfy the market requirements of large capability and memory devices with high speed [1,2,3,4,5]. The thin small outline package (TSOP), as a novel surface mount technique, has many advantages, such as being small in size, possessing high reliability, the large capability of the package, a short period regarding the package, and low cost. Furthermore, it can be anticipated that this new package technique will be widely used in packages for semiconductor memory devices, for example, smart phones, iPads, new energy vehicles, digital cameras, communication equipment, unmanned aerial vehicles, solid state disks, smart appliances, and data storage [6,7,8,9]. A new high-speed, large-capability TSOP package with 48 leads has been successfully designed and fabricated in this paper.

## 2. Structure Design and Process Experiments

### 2.1. Structure Design

**Figure 1:**
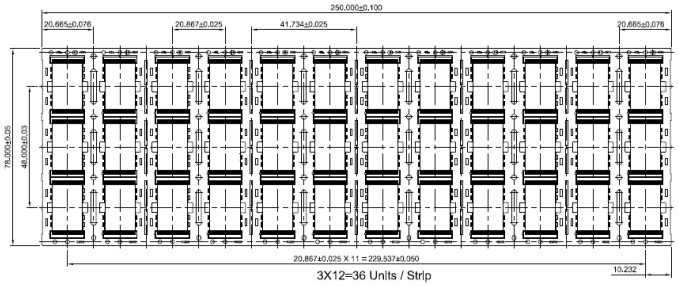
Structural sketch of leading frame TSOP48L.

The internal structure of the frame was designed by using long leading pins instead of base islands. In order to ensure that the long leading pins co-plane, the frame adhesive film was segmentally designed along the long leading pins. Then, the long leading pins were spliced with adhesive films co-planed together. The length of the colloidal was determined to be 8.40 mm, and the width of colloidal was 12.00 mm. The long leading pins were designed as concave shapes whose depth was 0.20 mm. The leading pins were partially coated with silver, and the size of chip was 7.909 mm × 7.503 mm. The lead frame composed of material C7025 1/2H was designed without base islands. The size of the external structure was length = 250 mm, width = 78 mm, and thickness = 0.127 mm, respectively, as shown in Figure 1, consisting of 3 × 12 strips.

### 2.2. Process Experiments

#### 2.2.1. Chip Backside Thinning

The thickness of chip wafer was reduced to the optimum value between 140 μm and 280μm, at which the internal stress and warp deformation of frame were minimum.

#### 2.2.2. Chip Scribing

In order to prevent chips from breaking or gaping, the pressure and speed of scribing chip were accurately controlled due to very narrow cutting blade pitch.

#### 2.2.3. Die Attaching

The high-density multi-row leading frame TSOP48L (3R × 12L) was designed by using long leading pins without base island in internal structure. The silver was plated locally. The thimble device with large curvature was designed to avoid chips breaking because of the stress release produced by scribing. The chips were placed at the frame without base islands. The conductive adhesive must be coated over the entire back of the chip to prevent the top side from being suspended, which could result in chip breakdown. 

#### 2.2.4. The 25 µm Precise Positioning Dice Attaching

**Figure 2:**
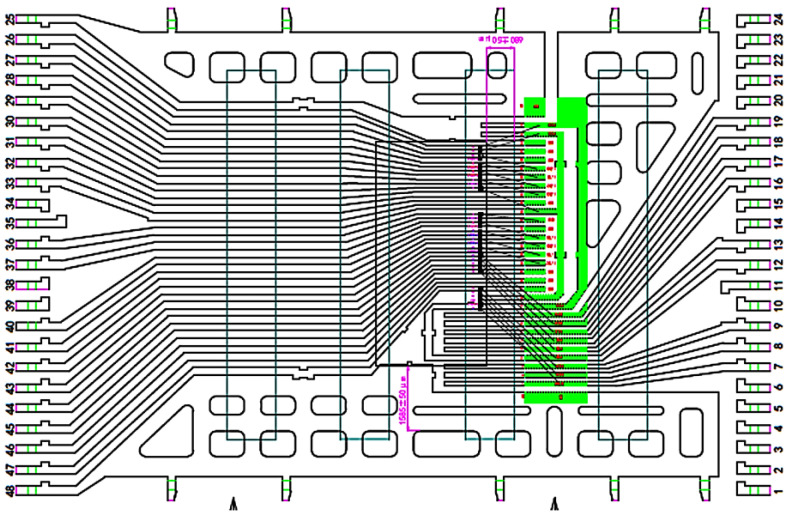
Layout of wire connection and chip bonding position (Light green represents the bonding pads, and dark green represents the glue strands).

TSOP48L chips were precisely placed at the internal pads of leading frame without island with maximum deviation less than ±25 μm. The distance between upside of chip and adhesive film is only 40 μm. If the deviation shift downward is ±50 μm, as shown Figure 2, the upside of chip may not contact the adhesive film, resulting in upside of chip rise to be suspended, poor contact, and chip breakdown during plastic sealing [2,10,11,12].

**Figure 3:**
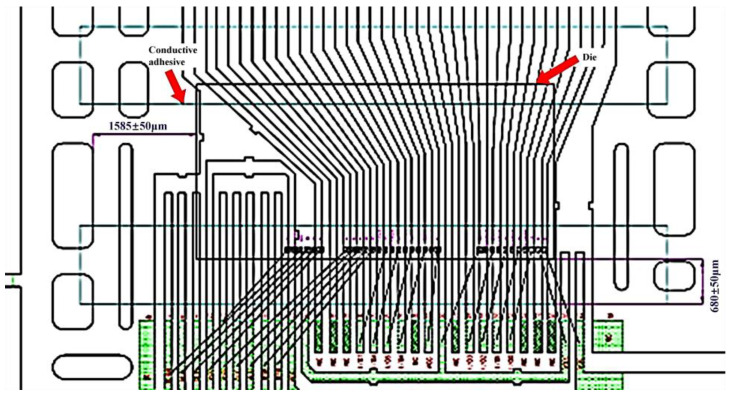
Adhesive film splicing position with chips (Light green represents the bonding pads, and dark green represents the glue strands).

The technologies of 25 µm precise positioning dice attaching were developed to ensure both upside and downside of chip can be pasted completely and reliably, as shown in Figure 3. An accurate positioning system was designed and fabricated on the base of original positioning equipment. In order to improve the resolution ratio and accuracy for positioning, the positioning scope was shrunk by using small-scale lens with high-rate magnification, together with combining image recognition techniques.

## 3. Key Technologies

### 3.1. Leading Frame Unit without Base Islands

**Figure 4:**
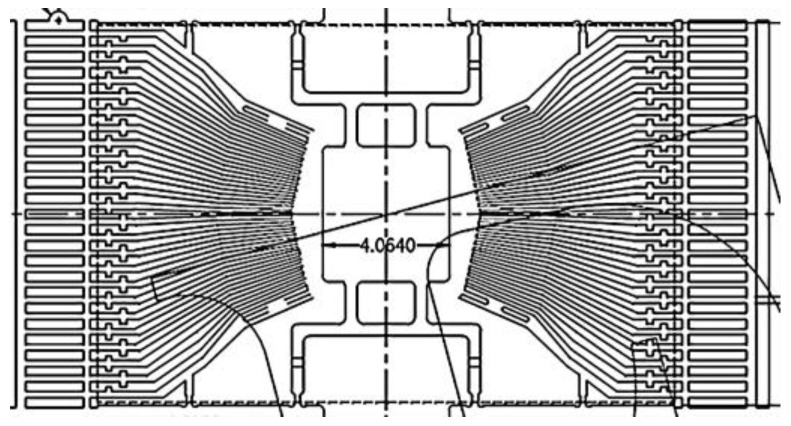
Traditional unit frame with base islands.

**Figure 5:**
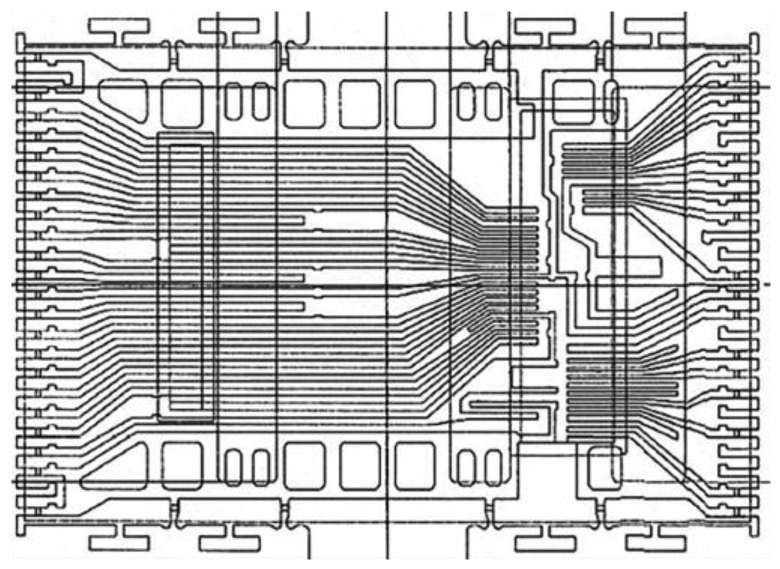
Unit frame without base islands.

A large part of the pad is located on the same side of the chips for TSOP48L. Traditionally, the frame island is located in the middle region. The bonding wires connecting the chip pads to the frame’s internal pins must cross over the chip, so the long pins were designed on this basis. In order to suit the package of memory chips whose pads are arranged at the one single side of it, a new lead frame structure without an island was designed based on COL (Chip on Lea) technology, substituting the traditional structure with base islands. The pads of the traditional chips are arranged around it, with the base island of the frame supporting and bearing the chips, as shown in Figure 4, which are electrically connected to the frame pins. Meanwhile, the memory chips have a special structure composed of many small storage units, so their pads are arranged on one side to increase the storage capacity. The traditional lead frame structure cannot be utilized for memory chips with pads located on the same side as the crisscrossing of the welding wires may result in short circuits. In order to make the pins located on the two sides lie at the same one, the long pins were designed such that one side of the pins can be led to the other one in a newly designed frame unit structure, as shown in Figure 5. The interlaced problem of the welding wire layout at the two sides was solved by pasting the chips on the long pins.

### 3.2. Co-Planarity of Leads in Frame

**Figure 6:**
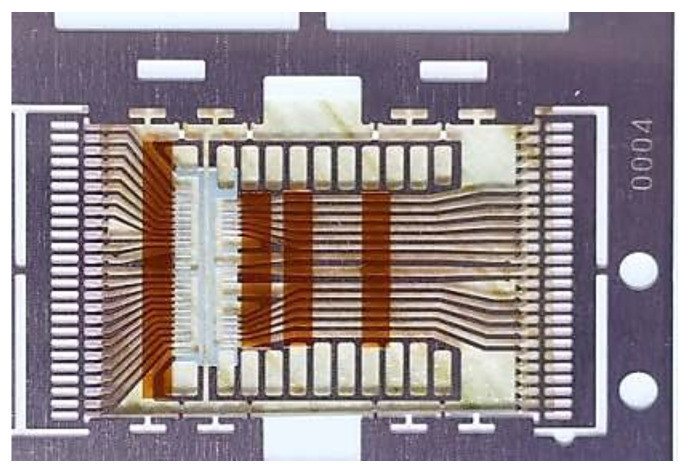
Diagram for adhesive film structure of lead pins in TSOP48L.

In the TSOP48L frame, the base islands were substituted by internal lead pins with a thin longitudinal shape. It is possible that warping deformation could occur, resulting in the co-planarity of the lead pins becoming worse. Therefore, three layers of adhesive film were designed to ensure the co-planarity of the internal lead pins via the adhesive force, as shown in Figure 6. 

### 3.3. Bonding Technique

The chips were fastened by using frame adhesive film and a DAE layer with 25 μm on the back of it. The bonding difficulty was increased since the elasticity of both the adhesive film and DAE layer may slide around. The bonding quality was heavily dependent on many parameters, such as ultrasonic power, heat pressure force, and time. The optimum bonding technological parameters for the chips fastened by the adhesive film and DAE layer, including USG = 140~160 mA, force = 35~45 g, and time = 18~22 ms, were obtained by the experiments. The plasma cleaning process for copper wire bonding was developed in this work. In order to make the frame surface smoother and combine more easily, the oxide on the surfaces of the frame and chips was removed via physical impacts by argon ions. The reliability of the products was significantly enhanced by the improvements in the frame surface evenness, the combination with copper balls, the effective bonding area of the inter-metallic compounds, and the binding cohesion of the copper wires with pads. The reliability was further improved by using coursing technology to enhance the adhesive force of the molding compound onto the frame, avoiding cracks occurring. The bonding radian must be less than 200 μm as the thickness of the whole plastic sealing body is only 1.00 mm, and the space under the curve is 250 μm. 

### 3.4. Plastic Sealing Technique

**Figure 7:**
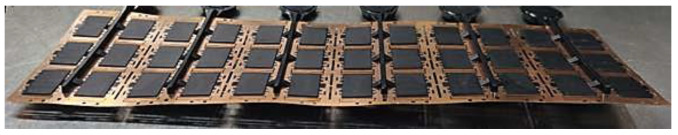
Diagrammatic sketch of product buckling after plastic sealing.

Crisscrossing and breaking of wires may occur due to the large number of wire lines present. It is a difficult problem to control the punch rate in a TSOP48L plastic package. To satisfy the requirement of a low punch rate, a plastic sealing material with excellent mobility and low viscosity was developed and prepared. The frame structure was designed as a multi-row matrix, being relatively soft, in which there are many units in every colloid strip, as shown in Figure 7. The lead frame may distort during the plastic package since the expansion coefficient difference between the frame and molding compound is about one order of magnitude, as well as having different internal heat stress. In order to prevent the frame being distorted, a pressure block was used during the period of high-temperature solidifying, avoiding the wires breaking and shorting. 

### 3.5. Anti-Warping Technique

**Figure 8:**
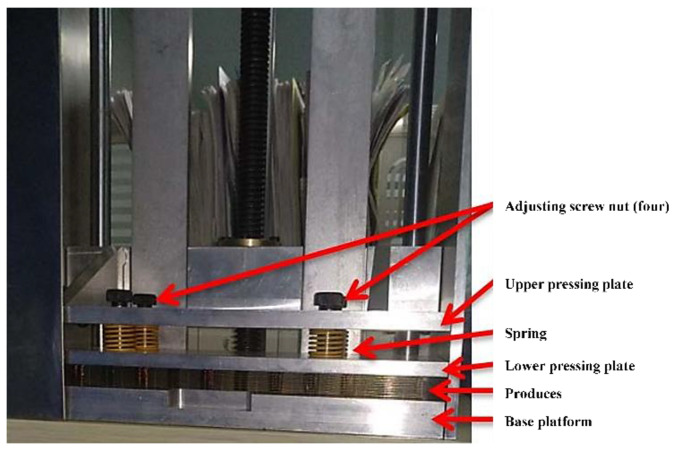
Illustrating sketch of anti-warping equipment for high-temperature solidification.

Due to the large size of the package capsule and the difference in the thermal expansion coefficients between the plastic compound and the copper frame, a change in the temperature from high to room level may cause the package capsule to warp, as shown in Figure 7. The anti-warping device in the high-temperature solidifying process was designed and fabricated. The plane pressure was increased to press the colloid tightly in the post-solidification stage at 175 ± 5 °C for 6.5 h to avoid or diminish the distortion or to bend upwards, as illustrated in Figure 8. 

### 3.6. Surface Roughness

In order to prohibit the plastic molding compound from the separating layer, a frame with the function of an anti-separating layer was designed and realized in this work. The frame surface was roughened to improve the adhesive strength of the molding compound on it. The base island was not electro-plated, and the silver-plating area was decreased to further enhance the adhesive strength. The distance between the cutting tool and the plastic sealing body was increased to prevent the external force from exerting on it in the process of the cutting reinforcement formation and to optimize the technological parameters of plastic injection with multiple segments. A special mechanism was designed in the frame island to prevent the plastic sealing material from overflowing. The black film overflowing was avoided effectively, having ensured the exposed area. 

## 4. Discussion

With the development of the function of consumer electronics and the trend of product intelligence, the application fields of IC memory are becoming increasingly widespread. The designed ultrathin TSOP48L, large in width with a high-speed IC memory package, will be widely applied in data operation, smart appliances, and intelligent manufacturing. The standardization of the IC package’s external dimensions and ultrathin characteristics was achieved through the developed TSOP48L package, which features a small outline based on the traditional SOP package and outline. It offers numerous advantages, including large size, high material utilization, low cost, high reliability, and a short production cycle. 

Consequently, it applies to digital cameras, smart TVs, automotive electronics, as well as high-end consumer electronic products. It is anticipated that this package may gain significant market traction and meet industry requirements.

## 5. Conclusions

The TSOP48L lead frame with high reliability was designed by not using an island, the chip attaching to the frame without an island, and pressure welding techniques were developed. The anti-warping and anti-bubble structures were designed for ultrathin plastic sealing bodies measuring 8.40 mm × 12.00 mm × 1.00 mm and 18.40 mm × 12.00 mm × 1.00 mm, respectively. The process parameters for chip thinning, die bonding, and plastic packaging were optimized. The complete processes for the TSOP48L package product with low cost and high reliability were developed. The package profile, frame unit layout, island structure, pin distribution, and the structure without an island for the novel TSOP package with 48 pins were designed and researched, indicating notable progress in addressing these challenges. Many technological problems, such as a reduction in the thickness of the ultrathin wafers, IC scribing, chip sticking, plastic sealing, and the IC trim/form, were studied and solved. The novel TSOP48L package technologies involved aspects including a multi-row matrix frame without a base island structure, long pins, pin isolation locking, different pin interconnections with the same common ground wire, the co-planarity of the long pin, multichip simultaneous loading, bonding and solidification at one time, and multichip stacking bonding. The interconnection welding between the multilayer chip stacking, the improvement in the plastic packaging quality, and the filling of large-size colloid products were achieved. Many technical problems were successfully resolved during the development of the TSOP48L package, including chip spallation, a cavity in the plastic sealing body, overflow control, chip cracks, silver alloy wire bonding, inter-connection welding, as well as chip delamination.

## Data Availability

All data available in the article.

## References

[1] Jessie D., Larson L. (2003). An improved leaded small outline package and equivalent circuit. IEEE Microw. Wirel. Compon. Lett..

[2] Priyabadini S., Sterken T., Van Hoorebeke L., Vanfleteren J. (2013). 3-D stacking of ultrathin chip packages: An innovative packaging and interconnection technology. IEEE Trans. Compon. Packag. Manuf. Technol..

[3] Yeo S.M., Yow H.K., Yeoh K.H., Azenal S.N.F.M. (2023). Elimination of Die-Pop Defect by Vacuum Reflow for Ultrathin Die with Warpage in Semiconductor Packaging Assembly. IEEE Trans. Reliab..

[4] Souriau J.-C., Poulain C., Castagné L., Ladner C., Hilt T., Franiatte R., Mermin D., David N. (2023). Flexible Hybrid Electronics Including Ultrathin Strain Sensors or Radio Frequency Identification Dies Manufactured on Wafer Silicon Carrier. IEEE Trans. Compon. Packag. Manuf. Technol..

[5] Bravin J., Burggraf J., Pichler M., Jung S., Brandl E. (2020). Laser Debonding of Ultrathin Wafers and Packages: A Technology for the New Generation of Electronic Devices. ECS Meet. Abstr..

[6] Fu L., Guo Q. (2010). Development of an ultra-small micro drill bit for packaging substrates. Circuit World.

[7] Ye G., Jiang M., Xue S., Ma W., Dai L. (2018). On the instability of chip flow in high-speed machining. Mech. Mater..

[8] Huh S.-H., Kim K.-D., Kim K.-S., Jang J.-S. (2012). A novel high-speed shear test for lead-free flip chip packages. Electron. Mater. Lett..

[9] Jiang N., Liu H., Zou J., Guo C., Li W., Shi M., Yang B., Liu Y., Guo B. (2021). Packaging design for improving the uniformity of Chip scale package (CSP) LED luminescence. Microelectron. Reliab..

[10] Kim M.S., Pulugurtha M.R., Sundaram V., Tummala R.R., Yun H. (2018). Ultrathin high-Q 2-D and 3-D RF inductors in glass packages. IEEE Trans. Compon. Packag. Manuf. Technol..

[11] Cho C.-L., Kao H.-L., Chang L.-C., Wu Y.-H., Chiu H.-C. (2019). Inkjet-printed vertical interconnects for ultrathin system-on-package technology. Surf. Coat. Technol..

[12] Singh B., Menezes G., McCann S., Jayaram V., Ray U., Sundaram V., Pulugurtha R., Smet V., Tummala R. (2017). Board-level thermal cycling and drop-test reliability of large, ultrathin glass BGA packages for smart mobile applications. IEEE Trans. Compon. Packag. Manuf. Technol..

